# The Examination of the Relationship Between Death Anxiety, Psychological Resilience and Religious Attitude Levels of Cardiology Patients with and without Myocardial Infarction in Turkey

**DOI:** 10.1007/s10943-025-02294-7

**Published:** 2025-03-28

**Authors:** Gülhan Yiğitalp, Rojda Bürçün

**Affiliations:** 1 Department of Nursing, Diyarbakır Ataturk Faculty of Health Sciences, Dicle University, Diyarbakır, Turkey; 2 Dicle University Heart Hospital, Diyarbakır, Turkey

**Keywords:** Cardiology patients, Myocardial infarction, Death anxiety, Resilience, Religious attitude

## Abstract

The study was conducted to investigate death anxiety, psychological resilience, religious attitude levels, and related factors in cardiology patients with and without myocardial infarction (MI). This descriptive and cross-sectional study was conducted with 500 cardiology patients (250 with and 250 without MI) in Turkey. The Personal Information Form, Turkish Death Anxiety Scale, Resilience Scale for Adults, and Ok-Religious Attitude Scale were used in the collection of the data. No statistically significant differences were detected between death anxiety, psychological resilience, and religious attitude levels in the two patient groups. According to the regression analysis results, retired people showed significantly higher levels of psychological resilience compared to other occupational groups, regular users of medication compared to non-users and sometimes regular users, and those who did not do regular physical activity compared to those who did not do any physical activity (*p* < 0.05). Psychological resilience decreased as the duration of illness increased (B = − 0.360; *p* = 0.001). Death anxiety and religious attitude had no significant effect on psychological resilience (B = − 0.070; *p* = 0.132; B = − 0.240; *p* = 0.192, respectively). Programs must be developed to reduce death anxiety and increase the psychological resilience of all cardiology patients, and religious coping methods must be included in these programs.

## Introduction

Cardiovascular diseases (CVD) are the leading cause of mortality globally. It was reported that 17.9 million people died from CVDs in 2019, which accounts for about a third of all deaths and 85.0% of these deaths occurred because of myocardial infarction (MI) and stroke. More than three-quarters of CVD deaths occur in low- and middle-income countries and pose a serious threat (WHO, 2021). When deaths in Turkey are analyzed according to their causes, circulatory system diseases take the first place with 36.8 and 39.1% of deaths from circulatory system diseases are caused by ischemic heart disease, 22.2% by cerebrovascular diseases, 25.7% by other heart diseases, and 7.9% by hypertensive diseases (TUIK, 2020). Cardiovascular diseases also cause 300.000 MI and 125.000 deaths every year in Turkey (TKD, 2020).

Psychological health problems occur in patients with heart disease as well as physical health problems. One of the psychological problems is death anxiety, which is defined as a natural reaction that reminds individuals of death and poses a threat to them (Soleimani et al., 2020). Death is an important factor that directs the thoughts and behaviors of individuals. Individuals finding themselves close to death, having a terminal illness, or facing the death of their relatives and loved ones may create fear for them. In a previous study, it was found that patients with MI had higher death anxiety than healthy individuals (Çaglar & Kaçer, 2022). In a comparative study that was conducted with patients with MI, cancer patients, and healthy people, it was reported that the patient group with MI experienced higher death anxiety than other groups (Şahan et al., 2018). In another study that was conducted with heart failure patients, it was reported that death anxiety was at a moderate level (Soleimani et al., 2020).

Psychological resilience and religious attitudes have important contributions to coping with death anxiety, which is one of the negative psychological conditions experienced by individuals with CVD. Psychological resilience is the ability to stay healthy and strong when faced with adverse situations (Liu et al., 2018). Psychological resilience has been found to have positive impacts on physical and mental health, as well as reduce the negative impacts of emotional stress. It was reported that resilience acts as a stress buffer for cardiovascular and metabolic diseases (Lehrer et al., 2020). It was reported that the recovery process of patients with high psychological resilience was better in patients with coronary heart disease (Chan et al., 2006). It was reported in many studies that sociodemographic characteristics such as age, gender, education, employment status, and duration of illness are also associated with resilience (Nouri-Saeed et al., 2015; Razaghpoor et al., 2021; Sakal & Sakal, 2024).

Religious belief is defined as a source of support in coping with negative situations (Böell et al., 2016). Religiosity is an important factor in the health of society and might affect the health behaviors of individuals with CVD (Elhag et al., 2022). It was reported that higher levels of religiosity and/or spirituality may be associated with better quality of life in patients with CVD (Abu et al., 2018). Although many studies report that individuals with a religious belief have better health outcomes, some studies show that they have a negative or no effect on the contrary (Mishra et al., 2017). Also, having a religious belief has a positive effect on resilience (Böell et al., 2016; Yıldırım & Gürsu, 2021). It was argued that the resilience levels of heart patients who stated that they were religious were higher (Bang et al., 2013). Having stronger religious beliefs was associated with lower death anxiety in individuals with heart disease (Soleimani, et al., 2020). No statistically significant relationships were detected between religious belief and death anxiety in patients with MI (Soleimani, et al., 2018).

CVDs are among the leading causes of mortality in Turkey. MI is also one of the CVDs that often results in death. Demonstrating death anxiety, psychological resilience and religious attitude levels in patients with CVD and/or MI will make an important contribution to the care and practices to be given to such patients. The present study will contribute to the literature because it is the first to examine whether there is a difference in terms of these variables in cardiology patients with and without MI. The study was conducted to investigate death anxiety, psychological resilience and religious attitude levels, and related factors in cardiology patients with and without myocardial infarction.

The study sought answers to the following questions.

1. What is the level of death anxiety, psychological resilience and religious attitudes of cardiology patients with or without myocardial infarction?
2. Do sociodemographic characteristics affect death anxiety, psychological resilience, and religious attitude levels of cardiology patients with or without myocardial infarction?
3. Is there a relationship between death anxiety and resilience in cardiology patients with or without myocardial infarction?
4. Is there a relationship between death anxiety and religious attitude in cardiology patients with or without myocardial infarction?
5. Is there a relationship between religious attitude and psychological resilience of cardiology patients with or without myocardial infarction?

## Methods

### Participants

The population of this descriptive and cross-sectional study consisted of cardiology patients who were aged 18 years and older, hospitalized in Diyarbakır Dicle University Cardiac Hospital in the Southeastern Anatolia Region of Turkey. With a total bed capacity of 204, this hospital is the first and only heart disease hospital in the Southeastern Anatolia Region and the largest in the Middle East. The convenience sampling method was used in the study. The cardiology patients, who were hospitalized in the heart disease hospital between July and December 2020, who volunteered to participate in the study and met the study criteria, were included in the study. The alpha value was found to be 0.05 using the G*Power program version 3.1.9.7, and the sample size was calculated as 134 to obtain 95% power with an effect size of 0.3 (Faul et al., 2007). The study was completed with a total of 500 patients (250 with and 250 without MI) to increase the power of the study.

*Inclusion criteria of the study*: Being diagnosed with CVD and/or MI by a specialist doctor, being hospitalized in the hospital for at least 24 h, having stable vital signs, being 18 years old and over, having the ability to understand and speak Turkish, volunteering to participate in the study, and having physical and mental competence.

### Data Collection Tools

The data were collected with four main forms/scales; 1. Personal Information Form, 2. Turkish Death Anxiety Scale (TDAS), 3. Adult Resilience Scale, and 4.Ok-Religious Attitude Scale.

#### Personal Information Form

This form was created by the researchers and included information on MI (yes/no), age (18–50, 51–64, 65–74, 75 and over), gender (female/male), educational status (illiterate, primary school, secondary school, high school and over), marital status (married, single, widowed), economic status (good, moderate, poor), occupation (unemployed, retired, civil servant, employee, tradesman, farmer), smoking and alcohol use (yes, no, given up), regular use of medications (Yes, No, Sometimes), presence of other chronic diseases (yes/no), physical activity (didn’t, doing regularly, not regular), height, weight, and disease. It also includes demographic and health/illness-related questions such as the duration of the disease, the presence and death of heart disease in the family, and the presence of a caregiver (yes/no).

#### Turkish Death Anxiety Scale (TDAS)

It is a 20-item scale that was developed by Sarıkaya and Baloğlu (2016). There are three sub-dimensions of death: uncertainty, contemplation and witnessing of death, and suffering. The scale items were prepared in a 5-point Likert form. Scale items are answered as “never, rarely, sometimes, often, and always.” The scale is scored between 0 and 80, and high scores indicate high death anxiety. Scores between 0 and 29 indicate low death anxiety, scores between 30 and 59 indicate moderate death anxiety, and scores between 60 and 80 indicate high death anxiety. The total Cronbach alpha coefficient of the scale was found to be 0.95 (Sarıkaya & Baloğlu, 2016).

#### Psychological Resilience Scale for Adults (PRSA)

PRSA was developed by Friborg et al. (2005) and was translated into Turkish by Basım and Çetin (2011), and its validity and reliability were established. There are 33 items on the scale, which has a total of six dimensions. In the scale, “structural style” (3, 9, 15, 21) and “perception of the future” (2, 8, 14, 20) have 4 items, “family adjustment” (5, 11, 17, 23, 26, 32), “self-perception” (1, 7, 13, 19, 28, 31), and “social competence” (4, 10, 16, 22, 25, 29) have 6 items each, and “social resources” (6, 12, 18, 24, 27, 30, 33) are measured with 7 items. The evaluation of the scale items was released as in the original study. If it is desired to increase psychological resilience as the scores increase in the Likert format, the answer boxes must be evaluated as 1, 2, 3, 4, and 5 from left to right. If this opinion is taken into account, questions 1–3–4–8–11–12–13–14–15–16–23–24–25–27–31 and 33 will be reverse questions in the scale. If it is desired to increase psychological resilience as the scores decrease, answer boxes will be evaluated as 5, 4, 3, 2, 1 and the reverse questions would then questions 2–5–6–7–9–10–17–18–19–20–21–22–26–28–29–30 and 32. As the scores increased, the preference for increasing psychological resilience was chosen in this study. The total Cronbach Alpha coefficient of the scale was 0.86 (Basım & Çetin, 2011).

#### Ok-Religious Attitude Scale

The Religious Attitude Scale, which was developed by Ok (2011), has a 5-point Likert-style and consists of 8 items and 4 sub-dimensions (cognition, emotion, behavior, and relationship). The scale is answered by marking one of the options “I strongly disagree,” “I slightly agree,” “I somewhat agree,” “I mostly agree,” and “I agree completely.” There are 6 positive and 2 negative items on the scale. The scale is evaluated between 8 and 40 points. A high score indicates a high religious attitude level of individuals, and a low score indicates a low religious attitude level. The Cronbach’s Alpha coefficient of the scale was found to be 0.90 for the total score (Table 1).

**Table 1:** Ok-Religious Attitude Scale

1	I feel there is no need for religion (-)
2	I feel religion does more harm than good to people (-)
3	I feel moved when I listen to religious chanting/reciting such as Ezan, prayer or Qur’anic verses
4	I really enjoy when I take part in religious activities
5	I check that I am living my life in line with religious values
6	I try to put my religion into practice in my life
7	I feel that God helps me when life is difficult
8	I feel that God is very close to me

### Data Collection

The patients who were hospitalized in cardiology clinics were informed about the purpose of the study, and their consent was obtained. Those who agreed to participate in the study and met the inclusion criteria were asked to fill out a questionnaire and those who had difficulties in filling out the questionnaire were assisted by the researchers. The data were collected by face-to-face interview method. A total of 630 patients were interviewed, of which 583 patients met the inclusion criteria. Among these patients, the study was completed with a total of 500 patients who accepted the study (250 MI, 250 MI). The response rate was determined as 85.7%. The average completion time of the questionnaire was 25–30 min.

### Ethical Approval

The study was performed in line with the Helsinki Declaration Guideline. The study was approved by the Dicle University Faculty of Medicine Non-Invasive Clinical Research Ethics Committee (07.05.2020-161). Necessary legal permissions were obtained from the Chief Physician of Dicle University (08.07.2020-65468). Informed consent was obtained from all participants who volunteered to participate in the study and they were assured that their privacy would be protected.

### Statistical Analysis

The data were analyzed by using the statistical program SPSS 20.0 (SPSS Inc., Chicago, IL, USA). Descriptive statistics (means and percentages) were used in the analysis of the data, the chi-square test was used in the relationship between categorical variables, kurtosis, skewness, and Shapiro–Wilk tests from normality tests, Mann–Whitney U test was used in the relationship between scales and categorical variables, Kruskal–Wallis test was used, and the Spearman Correlation and Multiple Linear Regression was used in the relationship between scales. The Mann–Whitney U test was used as a post hoc procedure to determine the source of the difference resulting from the Kruskal–Wallis test. All findings were tested at the 0.05 significance level.

## Results

A total of 50.4% of the patients who were included in the study were over 65 years old and 57.8% of them were male, 34.8% were illiterate, 84.2% were married, 25.4% have good economic conditions, 39.6% are unemployed. A total of 28.8% were current smokers, 91.4% did not drink alcohol, 48.4% were overweight, 21.4% were obese, and 61.4% did not do physical activity. A total of 53.8% of the patients had heart disease in their family, 28.8% had someone who died of heart disease in their family, and 96.4% had someone helping them.

In the present study, a statistically significant difference was detected between cardiology patients with and without MI in terms of gender (*p* = 0.037), education status (*p* = 0.037), marital status (*p* = 0.013), occupation (*p* = 0.001), regular use of medications (*p* = 0.001), and physical activity (0.017) (Table 2).

**Table 2:** General characteristics of patients with and without Myocardial Infarction

Characteristics		With MI (*n* = 250)	Without MI (*n* = 250)	Total	χ^2^/p*
		n(%)	n(%)	n(%)	
*Age*					
18–50		55 (53.4)	48 (46.6)	103 (100.0)	2.363
51–64		77 (53.1)	68 (46.9)	145 (100.0)	0.501
65–74		61 (45.2)	74 (54.8)	135 (100.0)	
75 and over		57 (48.7)	60 (51.3)	117 (100.0)	
*Gender*					4.338
Female		94 (44.5)	117 (55.5)	211 (100.0)	**0.037**
Male		156 (54.0)	133 (46.0)	289 (100.0)	
*Educational status*					
Illiterate		84 (48.3)	90 (51.7)	174 (100.0)	8.427
Primary school		82 (49.4)	84 (50.6)	166 (100.0)	**0.037**
Secondary school		64 (60.4)	42 (39.6)	106 (100.0)	
High school and over		20 (37.0)	34 (63.0)	54 (100.0)	
*Marital status*					
Married		222 (52.7)	199 (47.3)	421 (100.0)	0.683
Single		11 (42.3)	15 (57.7)	26 (100.0)	**0.013**
Widowed		17 (32.1)	36 (67.9)	53 (100.0)	
*Economic status*					
Good		67 (52.8)	60 (47.2)	127 (100.0)	0.540
Moderate		138 (49.3)	142 (50.7)	280 (100.0)	0.763
Poor		45 (48.4)	48 (51.6)	93 (100.0)	
*Occupation*					
Unemployed		81 (40.9)	117 (59.1)	198 (100.0)	21.505
Retired		24 (40.0)	36 (60.0)	60 (100.0)	**0.001**
Civil servant		22 (51.2)	21 (48.8)	43 (100.0)	
Employee		41 (57.7)	30 (42.3)	71 (100.0)	
Tradesman		35 (68.6)	16 (31.4)	51 (100.0)	
Farmer		47 (61.0)	30 (39.0)	77 (100.0)	
*Smoking*					
Still smoking		80 (55.6)	64 (44.4)	144 (100.0)	2.502
Never smoked		103 (47.9)	112 (52.1)	215 (100.0)	0.286
Given up		67 (47.5)	74 (52.5)	141 (100.0)	
*Drinking alcohol*					
Yes		1 (25.0)	3 (75.0)	4 (100.0)	1.661
No		227 (49.7)	230 (50.3)	457 (100.0)	0.436
Given up		22 (56.4)	17 (43.6)	39 (100.0)	
*Duration of illness*					
less than 1 year		16(39,0)	25(61,0)	41(100.0)	4.236 0,237
1–5 years		105(47,5)	116(52,5)	221(100.0)	
6–10 years		103(54,5)	86(45,5)	189(100.0)	
more than 10 years		26(53,1)	23(46,9)	49(100.0)	
*BMI*					
Weak		6 (42.9)	8 (57.1)	14 (100.0)	0.526
Normal		68 (49.6)	69 (50.4)	137 (100.0)	0.913
Overweight		124 (51.2)	118 (48.8)	242 (100.0)	
Obese		52 (48.6)	55 (51.4)	107 (100.0)	
*Regular use of medications*					
Yes		69 (39.0)	108 (61.0)	177 (100.0)	14.511
No		73 (52.5)	66 (47.5)	139 (100.0)	**0.001**
Sometimes		108 (58.7)	76 (41.3)	184 (100.0)	
*Another chronic disease*					
Yes		143 (50.7)	139 (49.3)	282 (100.0)	0.130
No		107 (49.1)	111 (50.9)	218 (100.0)	0.718
*Physical activity*					
Didn’t		169 (55.0)	138 (45.0)	307 (100.0)	8.116
Doing regularly		22 (41.5)	31 (58.5)	53 (100.0)	**0.017**
Not regular		59 (42.1)	81 (57.9)	140 (100.0)	
*Presence of heart disease in the family*					
Yes		145 (53.9)	124 (46.1)	269 (100.0)	3.548
No		105 (45.5)	126 (54.5)	231 (100.0)	0.060
*Death due to heart disease in the family*					
Yes	72 (50.0)		72 (50.0)	144 (100.0)	0.001
No	178 (50.0)		178 (50.0)	356 (100.0)	1.000
*Presence of someone helping*					
Yes	237 (49.2)		245 (50.8)	482 (100.0)	3.688
No	13 (72.2)		5 (27.8)	18 (100.0)	0.055

*Statistically significant data are given in bold

Patients’ Death Anxiety Total score was found to be 30.45 ± 10.89, Psychological Resilience total score was 100.88 ± 11.95, Religious Attitude total score was 36.40 ± 2.79, and their medians were 30.00, 99.00, and 37.00, respectively (Table 3).

**Table 3:** Scores of cardiology patients from the scales

Variables	n	Min–Max	Median	Mean ± SD
Death anxiety	500	0–80	30.00	30,45 ± 10,89
Psychological resilience	500	74–160	99.00	100.88 ± 11.95
Religious attitude	500	25–40	37.00	36.40 ± 2.79

No statistically significant differences were detected between death anxiety, psychological resilience, and religious attitude levels between cardiology patients with and without MI (*p* > 0.05). No statistical differences were found between the sociodemographic characteristics of the patients and their death anxiety scores (*p* > 0.05). There was a statistically significant difference between gender (*p* = 0.013), occupation (*p* = 0.002), duration of illness (*p* < 0,001), regular use of medications (*p* = 0.014), physical activity (*p* = 0.028), and psychological resilience levels. Men compared to women, retirees compared to other occupational groups, those who used their medication regularly compared to those who did not use and sometimes regularly used, and those who did not do regular physical activity compared to those who did not do any physical activity. As the duration of illness increased, psychological resilience decreased. Statistically significant differences were detected between age (*p* = 0.012), disease duration (*p* < 0,001), presence of heart disease in the family (*p* = 0.003), and religious attitude levels. As the age and duration of illness increased, the level of religious attitude decreased. The level of religious attitudes was found to be higher in those who did not have heart disease in the family (Table 4).

**Table 4:** Comparison of death anxiety, psychological resilience and religious attitude levels of cardiology patients in terms of sociodemographic characteristics

Characteristics (*n* = 500)	Death Anxiety*		Psychological Resilience*		Religious Attitude*	Mean (SD)
	Median (Min–Max)	Mean (SD)	Median (Min–Max)	Mean (SD)	Median (Min–Max)	
*Have MI*						
Yes (*n* = 250)	30 (4–77)	30.37 ± 8.35	98 (76–160)	99.49 ± 9.69	37 (28–40)	36.38 ± 2.71
No (*n* = 250)	30 (0–80)	30.53 ± 12.97	100 (74–156)	102.28 ± 13.73	37 (25–40)	36.43 ± 2.88
^a^p	0.846		0.124		0.712	
*Age*	65.00 (18–95)	63.30 ± 14.13				
^b^p	0.073/0.104		− 0.038/0.392		− **0.112/0.012**	
*Gender*						
Female (*n* = 211)	30 (0–77)	30.91 ± 10.93	98 (74–150)	99.09 ± 10.03	37 (25–40)	36.17 ± 2.96
Male (*n* = 289)	29 (0–80)	30.11 ± 10.88	100 (78–160)	102.2 ± 13.04	37 (28–40)	36.58 ± 2.66
^a^p	0.457		**0.013**		0.187	
*Educational status*						
Illiterate (*n* = 174)	30 (0–74)	30.43 ± 9.73	98 (74–155)	99.87 ± 11.59	37 (28–40)	36.49 ± 2.95
Primary school (*n* = 166)	30 (0–80)	30.92 ± 11.89	99.5 (78–139)	101.13 ± 11.19	37 (28–40)	36.58 ± 2.74
Secondary school (*n* = 106)	30 (0–77)	29.86 ± 9.96	99 (78–160)	100.61 ± 11.30	36 (28–40)	36.08 ± 2.59
High school and over (*n* = 54)	28.5 (8–75)	30.24 ± 13.06	101 (83–156)	103.96 ± 15.84	37 (25–40)	36.24 ± 2.82
^c^p	0.938		0.427		0.167	
*Marital status*						
Married (*n* = 421)	30 (0–80)	30.29 ± 9.80	99 (76–160)	100.97 ± 11.88	37 (28–40)	36.48 ± 2.69
Single (*n* = 26)	28.5 (8–75)	30.12 ± 13.99	99.5 (78–130)	99.08 ± 10.46	37 (25–40)	36.73 ± 3.45
Widowed (*n* = 53)	31 (0–77)	31.85 ± 16.35	97 (74–150)	101.13 ± 13.31	36 (28–40)	35.62 ± 3.18
^c^p	0.542		0.847		0.095	
*Economic status*						
Good (*n* = 127)	30 (7–75)	30.39 ± 8.50	97 (78–139)	99.17 ± 10.49	37 (28–40)	36.39 ± 2.54
Moderate (*n* = 280)	30 (0–80)	30.28 ± 12.10	100 (74–160)	102.18 ± 13.53	37 (25–40)	36.5 ± 2.87
Poor (*n* = 93)	29 (4–70)	31.04 ± 10.02	99 (84–131)	99.34 ± 7.61	37 (28–40)	36.13 ± 2.92
^c^p	0.878		0.104		0.540	
*Occupation*						
Unemployed (*n* = 198)^1^	30 (0–80)	30.63 ± 11.61	99 (74–160)	99.73 ± 10.74	37 (28–40)	36.35 ± 2.85
Retired (*n* = 60)^2^	32.5 (0–68)	31.68 ± 14.49	105.5 (83–156)	108.03 ± 16.33	36 (28–40)	35.95 ± 2.91
Civil Servant (*n* = 43)^3^	29 (10–75)	29.63 ± 9.29	98 (78–141)	98.77 ± 11.44	37 (25–40)	36.58 ± 3.11
Employee (*n* = 71)^4^	30 (6–66)	31.14 ± 9.85	99 (83–153)	100.56 ± 11.76	37 (30–40)	36.42 ± 2.44
Tradesman (*n* = 51)^5^	28 (4–62)	29.59 ± 9.23	100 (78–131)	101.06 ± 10.68	37 (30–40)	37.02 ± 2.53
Farmer (*n* = 77)^6^	29 (0–63)	29.42 ± 8.38	97 (83–155)	99.66 ± 10.47	37 (28–40)	36.38 ± 2.85
^c^p	0.357		**0.002**	**2 > 1,3,4,5,6**	0.392	
*Smoking*						
Still smoking (*n* = 144)	30 (0–75)	30.22 ± 10.47	100 (78–160)	101.83 ± 13.05	37 (29–40)	36.58 ± 2.55
Never smoked (*n* = 215)	30 (0–74)	29.53 ± 10.21	98 (74–156)	99.72 ± 11.03	37 (25–40)	36.29 ± 2.95
Given up (*n* = 141)	31 (3–80)	32.09 ± 12.16	100 (78–152)	101.70 ± 12.08	37 (28–40)	36.4 ± 2.81
^c^p	0.241		0.171		0.892	
*Drinking alcohol*						
Yes/Given up (*n* = 43)	28 (8–63)	29.91 ± 9.41	99 (78–126)	99.30 ± 10.59	37 (30–40)	36.02 ± 2.56
No (*n* = 457)	30 (0–80)	30.50 ± 11.03	99 (74–160)	101.04 ± 12.08	37 (25–40)	36.44 ± 2.82
^a^p	0.368		0.529		0.242	
*Duration of Illness*	5.00 (01–21)	5,69 ± 4.85				
^b^p	0,083/0,065		**− 0.174/0.000**		**− 0.172/0.000**	
*BMI*	27.31 (15.57–44.44)	26.92 ± 4.63				
^b^p	0.031/0.486		**− **0.054/0.225		0.041/0.363	
*Regular use of medications*						
Yes (*n* = 177)^1^	30 (0–80)	32.07 ± 15.04	100 (74–160)	104.5 ± 16.00	37 (25–40)	36.38 ± 3.08
No (*n* = 139)^2^	28 (0–63)	28.84 ± 8.96	99 (85–131)	99.45 ± 8.33	37 (29–40)	36.5 0 ± 2.71
Sometimes (*n* = 184)^3^	30 (5–63)	30.11 ± 6.41	98 (76–126)	98.49 ± 8.43	37 (29–40)	36.35 ± 2.57
^c^p	0.065		**0.014**	**1 > 2,3**	0.694	
*Another chronic disease*						
Yes (*n* = 282)	30 (0–80)	30.75 ± 10.48	99 (74–156)	101.05 ± 12.34	37 (28–40)	36.18 ± 2.94
No (*n* = 218)	30 (0–77)	30.06 ± 11.43	99 (78–160)	100.67 ± 11.46	37 (25–40)	36.70 ± 2.58
^a^p	0.952		0.890		0.091	
*Physical activity*						
Didn’t (*n* = 307)^1^	29 (0–80)	30.50 ± 10.32	98 (74–156)	99.46 ± 10.36	37 (28–40)	36.52 ± 2.87
Doing regularly (*n* = 53)^2^	30 (4–75)	30.49 ± 13.87	99 (83–153)	102.66 ± 13.14	37 (28–40)	36.06 ± 2.89
Not regular (*n* = 140)^3^	30 (0–62)	30.34 ± 10.95	100 (78–160)	103.35 ± 14.15	36.5 (25–40)	36.29 ± 2.59
^c^p	0.370		**0.028**	**3 > 1**	0.206	
*Presence of heart disease in the family*						
Yes (*n* = 269)	30 (0–77)	29.76 ± 9.76	99 (76–155)	100.30 ± 10.85	37 (28–40)	36.10 ± 2.78
No (*n* = 231)	30 (0–80)	31.26 ± 12.06	99 (74–160)	101.56 ± 13.12	37 (25–40)	36.76 ± 2.78
^a^p	0.805		0.671		**0.003**	
*Death due to heart disease in the family*						
Yes (*n* = 144)	30 (0–77)	30.60 ± 10.83	98 (78–156)	100.46 ± 11.41	37 (28–40)	36.08 ± 2.84
No (*n* = 356)	30 (0–80)	30.39 ± 10.94	99 (74–160)	101.06 ± 12.18	37 (25–40)	36.53 ± 2.77
^a^p	0.665		0.664		0.073	
*Presence of someone helping*						
Yes (*n* = 482)	30 (0–80)	30.44 ± 10.94	99 (74–160)	100.99 ± 12.08	37 (25–40)	36.42 ± 2.79
No (*n* = 18)	28 (22–63)	30.78 ± 9.86	98.5 (84–110)	98.06 ± 7.63	37 (30–40)	36.06 ± 2.90
^a^p	0.532		0.532		0.663	

^a^Mann–Whitney U Test, ^b^Spearman Correlation, ^c^Kruskal–Wallis Test, ^1>2–6^ Post hoc analysis

*Statistically significant data are given in bold

A statistically significant relationship was detected between the psychological resilience and religious attitude total scores of the patients in the correlation analysis. A very weak negative (r = − 0.090, *p* = 0.045) correlation was found between psychological resilience and religious attitude scores (Table 5).

**Table 5:** Correlations between scale mean scores

^a^Scales	Death anxiety scale	Resilience scale*	Religious attitude scale
Death anxiety scale	1		
Resilience scale	**− **.029	1	
Religious attitude scale	**− **.066	^**b**^**− .090**	1

^a^Spearman Correlation,

^b^The correlation is significant at the *p*:0.05 level (2-tailed)

*Statistically significant data are given in bold

The regression model established with some demographic characteristics and death anxiety affecting psychological resilience is given in Table 6. The model was found to be statistically significant (F = 6.445, *p* = 0,000). The rate of the model explaining the dependent variable is (Adj.R^2^ = 0,116). Durbin–Watson value (1.944) shows that there is no autocorrelation in the model. According to the regression model, although men had higher psychological resilience than women, no significant difference was found (B = 2.372; *p* = 0.93). Psychological resilience of retired people was significantly higher than the other groups (*p* < 0.05). Psychological resilience of regular medications users was significantly higher than non-users and sometimes users (*p* = 0.003; *p* = 0.000). Psychological resilience was significantly higher in those who did not do physical activity regularly than in those who did not do it at all (B = 2.365; *p* = 0.047). Psychological resilience decreased as the duration of illness increased (B = − 0.339; *p* = 0.001). Death anxiety had no significant effect on psychological resilience (B = − 0.070; *p* = 0.132).

**Table 6:** Death anxiety and the effect of some variables on psychological resilience

	B(%95 CI)	SE	Beta	t	p*	VIF
Constant	111.394 (106.047–116.742)	2.721		40.933	.000	
Gender-Male	2.372 (− 397–5.141)	1.409	.098	1.683	.093	1.917
Occupation (Ref: Retired)						
Occupation-Unemployed	**− **5.413 (**− **9.288–1.539)	1.972	**− **.222	**− **2.745	**.006**	3.681
Occupation-Civil Servant	**− **8.660 (**− **13.140–4.179)	2.280	**− **.203	**− **3.797	**.000**	1.618
Occupation-Employee	**− **7.566 (**− **11.459–3.673)	1.981	**− **.221	**− **3.818	**.000**	1.893
Occupation-Tradesman	**− **6.036 (**− **10.336–1.735)	2.189	**− **.153	**− **2.758	**.006**	1.737
Occupation-Farmer	**− **6.874 (**− **10.768–2.980)	1.982	**− **.208	**− **3.468	**.001**	2.025
Regular use of medications (Ref: Yes)						
Regular use of medications-No	**− **3.921 (**− **6.524–1.317)	1.325	**− **.147	**− **2.959	**.003**	1.395
Regular use of medications-Sometimes	**− **5.400 (**− **7.779–3.021)	1.211	**− **.218	**− **4.460	**.000**	1.349
Physical activity (Ref: Didn’t)						
Physical activity—Doing regularly	2.313 (**− **1.011–5.637)	1.692	.060	1.367	.172	1.073
Physical activity—Not regular	2.365 (.031–4.700)	1.188	.089	1.991	**.047**	1.127
Duration of Illness	**− **.339 (**− **.547–.130)	.106	**− **.137	**− **3.194	**.001**	1.045
Death anxiety scale	**− **.070 (**− **162–021)	.047	**− **.064	**− **1.510	.132	1.021

B: Unstandardized Coefficients, Beta: Standardized Coefficients, F = 6.445, *p* = 0.000, Adj.R^2^ = 0.116, SE = 11.240. Durbin–Watson:1.944

*Statistically significant data are given in bold

The regression model established with some demographic characteristics and religious attitude affecting psychological resilience is given in Table 7. The model was found to be statistically significant (F = 6.399, *p* = 0,000). The rate of explaining the dependent variable of the model is (Adj.R^2^ = 0,115). Durbin–Watson value (1.976) shows that there is no autocorrelation in the model. According to the regression model, although men had higher psychological resilience than women, no significant difference was found (B = 2.610; *p* = 0.66). Psychological resilience of retired people was significantly higher than the other groups (*p* < 0.05). Psychological resilience of regular medications users was significantly higher than non-users and occasional users (*P* = 0.006; *p* = 0.000). Psychological resilience was found to be significantly higher in those who did not do physical activity regularly than in those who did not do it at all (B = 2.350; *p* = 0.049). Psychological resilience decreased as the duration of illness increased (B = − 0.360; *p* = 0.001). Religious attitude had no significant effect on psychological resilience (B = − 0.240; *p* = 0.192).

**Table 7:** The effect of religious attitude and some variables on psychological resilience

	B(%95 CI)	SE	Beta	t	p*	VIF
Constant	117.614 (103.928–131.301)	6.966		16.885	.000	
Gender-Male	2.610 (**− **.168–5.388)	1.414	.108	1.846	.066	1.927
Occupation (Ref: Retired)						
Occupation-Unemployed	**− **5.123 (− 9.007–1.240)	1.977	**− **.210	**− **2.592	**.010**	3.694
Occupation-Civil Servant	**− **8.312 (− 12.798–3.826)	2.283	**− **.195	**− **3.641	**.000**	1.620
Occupation-Employee	**− **7.437 (− 11.334–3.539-	1.984	**− **.217	**− **3.749	**.000**	1.895
Occupation-Tradesman	**− **5.728 (− 10.040–1.417)	2.194	**− **.145	**− **2.611	**.009**	1.743
Occupation-Farmer	**− **6.667 (− 10.562–2.772)	1.982	**− **.201	**− **3.363	**.001**	2.023
Regular use of medications (Ref: Yes)						
Regular use of medications-No	**− **3.660 (**− **6.248–1.071)	1.317	**− **.137	**− **2.778	**.006**	1.377
Regular use of medications-Sometimes	**− **5.237 (**− **7.610–2.864)	1.208	**− **.211	**− **4.336	**.000**	1.341
Physical activity (Ref: Didn’t)						
Physical activity—Doing regularly	2.254 (**− **1.075–5.583)	1.694	.058	1.330	.184	1.075
Physical activity—Not regular	2.350 (.013–4.687)	1.190	.088	1.976	**.049**	1.128
Duration of Illness	**− **.360 (**− **.571–.150)	.107	**− **.146	**− **3.363	**.001**	1.064
Religious Attitude Scale	**− **.240 (**− **.601–.121)	.184	**− **.056	**− **1.306	.192	1.038

B: Unstandardized Coefficients, Beta: Standardized Coefficients, F = 6.399, *p* = 0.000, Adj.R^2^ = 0.115, SE = 11.246. Durbin–Watson:1.976

*Statistically significant data are given in bold

## Discussion

It was not investigated in previous studies whether there is a difference in terms of death anxiety, psychological resilience, and religious attitude levels of cardiology patients with and without MI. Since this study is the first in this field, the results obtained will make valuable contributions to the literature in this field.

Death anxiety and psychological resilience were found to be moderate, and religious attitudes were high in cardiology patients with and without MI in the present study. Similar results were obtained in other studies (Al Ali & Al Ramamneh, 2022; Lemos et al., 2016; Nouri-Saeed et al., 2015; Park et al., 2014; Tülüce & Kaplan Serin, 2024). In the present study, it is possible to argue that the death anxiety and psychological resilience of cardiology patients were moderate. Also, the high religious attitudes show the importance given to religious values in our country.

The results of the study showed that no statistically significant differences were detected between death anxiety, resilience, and religious attitude levels of cardiology patients with and without MI. Similarly, in a previous study conducted on patients with Takotsubo cardiomyopathy or a history of myocardial infarction, no difference was observed between the two patient groups in terms of physical and mental impacts (Olliges et al., 2020). This can be explained by the same mental and psychological impacts in all cardiology patients with and without MI since cardiovascular events create an equal burden for patients.

In many studies conducted with individuals with CVD, it was stated that men had higher resilience scores than women (Carvalho et al., 2016; Love et al., 2021; Malik & Afzal, 2015). This study also supports other studies. Because of the roles assumed in terms of gender in society, the way women and men express their feelings may change and men may be more reluctant to express their anxiety and fears (Soleimani et al., 2018). For this reason, men may have expressed that they are more resilient to show themselves as stronger.

In the present study, retirees had higher levels of psychological resilience when compared to other occupational groups. Contrary to our study results, it was found in a study conducted with patients with coronary heart disease in Jordan that working patients were more likely to “accept change positively and establish a secure relationship,” which is a dimension of resilience, than retired patients (Al Ali & Al Ramamneh, 2022). In another study that was conducted with patients with chronic diseases, no relationship was found between occupation and resilience (Böell et al., 2016). These contradictory findings may be because the study was conducted with different groups. It is thought that retirees may have used their own coping methods. More studies are needed to clarify the relationship between resilience with occupation.

It was also determined that the duration of illness affects resilience levels, and psychological resilience decreases as the duration of illness increases, in line with previous studies on individuals with cardiological disease (Razaghpoor et al., 2021). Fighting diseases for a long time can reduce the resilience of individuals.

Consistent with the findings of the study that was conducted with different patient groups in the current study (Freire de Medeiros et al., 2017), the psychological resilience levels of the patients who used their drugs regularly were found to be higher. Contrary to this, in a study that was conducted with patients with coronary heart disease in Jordan, patients who did not comply with drug therapy showed higher resilience than those who complied (Al Ali & Al Ramamneh, 2022). Resilience is the ability to adapt positively to living conditions (Sisto et al., 2019). Despite these different results, patients who score high on resilience may engage in health-promoting behaviors such as eating healthy, exercising, having health checkups, and complying with prescription drugs (Dale et al., 2014).

It was stated that physical exercise can increase resilience by regulating the hypothalamic–pituitary–adrenal axis to buffer the effect of daily stress (Zschucke et al., 2015). In the present study, the psychological resilience levels of the patients who did not do physical activity were lower. This finding is consistent with the findings of other studies in which resilience was found to be an important indicator of participation in health-promoting behaviors in patients with CVD (Shin et al., 2015). It was also consistent with the study stating that exercise duration in elderly patients with CVD was a predictor of resilience (Lee et al., 2020). It is possible to argue that physical activity is effective in increasing psychological resilience as well as having many beneficial impacts.

Although there was a positive relationship between age and religious attitude in other studies conducted with individuals with CVD (Ai et al., 2009; Soleimani et al., 2018). In the study, on the contrary, a negative correlation was detected between the two variables. Although it has been stated that older individuals have higher religious and spiritual tendencies, younger individuals who are more aware of conditions that threaten the life of the individual such as CVD may have shown a more spiritual and religious inclination.

In this study, as the duration of illness increased, religious attitudes decreased. It was reported that the disease duration does not affect the level of positive religious coping in patients with acute coronary syndrome (Celik et al., 2022). In studies that were conducted with other patient groups, for example, in a study conducted with hemodialysis patients, similar to our study, it was reported that as the duration of treatment increased, patients used more negative religious coping styles (Ayık & Yılmaz Karabulutlu, 2020), and unlike our study, positive religious coping levels were found to be higher as the duration of the disease increased in patients with breast cancer (Altıntaş, 2019). It is possible to argue that religious attitudes and religious coping levels may differ according to the characteristics of the patients as the duration of the disease increases in individuals with chronic diseases. These findings show that the relationship between the two variables may differ depending on the type of disease. In this study, individuals who lived with the disease for a long time may have weakened their religious beliefs because they believed that they could not fully recover.

In the present study, religious attitude levels were higher in those who did not have a family history of heart disease. Since having a family history of heart disease may indicate that there is a hereditary disease, they may be more likely to accept the disease and thus their religious tendencies may be weaker. On the other hand, people who do not have a family history of heart disease may be worried that only they have this disease, and thus their religious tendencies as a coping method might have increased.

The relationship between religious attitude, which is the dimension of mental well-being, and psychological resilience was investigated in many studies involving individuals with chronic health conditions. In these studies, a positive relationship was reported between religious attitude and resilience (Al Eid et al., 2020; Böell et al., 2016; Fradelos et al., 2018). In a previous study that was conducted with patients with heart failure in Iran, the results revealed a significant positive relationship between resilience and the religious dimensions of mental well-being (Razaghpoor et al., 2021). Although many studies indicate that individuals with a religious belief have better health outcomes, some studies report that they have a negative or no effect on the contrary (Mishra et al., 2017). This study surprisingly showed a negative relationship between the two variables, which can be explained by the fact that in this study, because of the higher average age of the patients, the elderly individuals had difficulty perceiving their situation, and their religious attitudes were lower than the younger ones, thus reflecting this situation on their resilience levels. Also, although their religious attitudes are high, patients may be inadequate in using it as a coping method. Life-threatening conditions such as CVD and MI may have affected patients’ resilience levels differently. In this study, no relationship was found between religious attitude and psychological resilience, and death anxiety. In the study conducted with patients who survived acute MI, no significant difference was found between death anxiety and religious belief (Soleimani et al., 2018).

### Limitations

The study provides valuable information on death anxiety, resilience, and religious attitude levels in cardiology patients in Turkey. However, the study had some limitations. The first was the limitations in establishing causal relationships because of the cross-sectional nature of the study. Then, the generalizability of the findings is limited because of the convenience sampling method used in the study. Thirdly, although the study was conducted voluntarily and patients were assured of the confidentiality of the information, they may not have provided clear information because of the sensitivity of their situation. Last but not least, the study was conducted with patients with similar cultural characteristics because the study was conducted in a single center.

## Conclusion and Recommendations

Death anxiety and psychological resilience of the patients were found to be moderate in the study, and their religious attitudes were high. No significant differences were detected between death anxiety, psychological resilience, and religious attitude levels between cardiology patients with and without MI. Men, retired people, those who used their medications regularly, and those who did not engage in regular physical activity had higher psychological resilience. Psychological resilience decreased as the duration of illness increased. Religious attitudes decreased with increasing age and duration of illness. Those who did not have a family history of heart disease had higher levels of religious attitudes. Also, death anxiety was not associated with psychological resilience and religious attitude levels. There was a negative correlation between the psychological resilience of the patients and their religious attitudes.

Since CVDs are common in society and threaten life, patients must be followed up and supported psychologically as well as their physical health checks. Rehabilitation programs must be developed and implemented with the help of psychiatrists and psychologists to reduce death anxiety and increase psychological resilience in all cardiology patients. Public healthcare nurses must also support patients in the implementation of these programs, especially after discharge. Although the religious attitude levels of the patients were found to be high in the present study, a negative relationship was found between psychological resilience and religious attitude, but it is known that there is a positive relationship between the two variables in many previous studies (Fradelos et al., 2018; Razaghpoor et al., 2021). For this reason, religious coping methods must be included in the programs developed. Based on the results of this study, in future studies, comparative studies must be planned using a control group on the relationship between death anxiety, resilience, and religious attitude in cardiology patients with and without IM.
